# Body mass index and mild cognitive impairment among rural older adults in China: the moderating roles of gender and age

**DOI:** 10.1186/s12888-021-03059-8

**Published:** 2021-01-23

**Authors:** Yemin Yuan, Jie Li, Nan Zhang, Peipei Fu, Zhengyue Jing, Caiting Yu, Dan Zhao, Wenting Hao, Chengchao Zhou

**Affiliations:** 1 Centre for Health Management and Policy Research, School of Public Health, Cheeloo College of Medicine, Shandong University, Jinan, 250012 China; 2 Department of Epidemiology, School of Public Health, Cheeloo College of Medicine, Shandong University, 44 Wenhuaxi Road, Jinan, 250012 Shandong China; 3 Manchester Institute for Collaborative Research on Ageing, Social Statistics, School of Social Sciences, The University of Manchester, Manchester, UK; 4 NHC Key Laboratory of Health Economics and Policy Research, Shandong University, 44 Wenhuaxi Road, Jinan, 250012 Shandong China

**Keywords:** Body mass index, Mild cognitive impairment, Older adults, Gender difference, Age difference

## Abstract

**Background:**

Evidence concerning the association between body mass index (BMI) and cognitive function among older people is inconsistent. This study aimed to investigate gender and age as moderators in association between BMI and mild cognitive impairment (MCI) among rural older adults.

**Methods:**

Data were derived from the 2019 Health Service for Rural Elderly Families Survey in Shandong, China. In total, 3242 people aged 60 years and above were included in the analysis. Multilevel mixed-effects logistic regression was used to examine the moderating roles of gender and age, then further to explore the relationship between BMI and MCI.

**Results:**

There were 601 (18.5%) participants with MCI. Compared with normal BMI group, low BMI group had a higher risk of MCI among older people [adjusted odds ratio (aOR) = 2.08, 95% confidence interval (CI): 1.26–3.44], women (aOR = 2.06, 95% CI: 1.35–3.12), or the older elderly aged ≥75 years old (aOR = 3.20, 95% CI: 1.34–7.45). This effect remained statistically significant among older women (aOR = 3.38, 95% CI: 1.69–6.73). Among older men, elevated BMI group had a higher risk of MCI (aOR = 2.32, 95% CI: 1.17–4.61) than normal BMI group.

**Conclusions:**

Gender and age moderated the association between BMI and MCI among Chinese rural older adults. Older women with low BMI were more likely to have MCI, but older men with elevated BMI were more likely to have MCI. These findings suggest rural community managers strengthen the health management by grouping the weight of older people to prevent the risk of dementia.

**Supplementary Information:**

The online version contains supplementary material available at 10.1186/s12888-021-03059-8.

## Background

Diseases related to cognitive impairment, especially dementia, are increasingly becoming prominent health threat and bring a huge burden in older people worldwide. In 2018, there were 50 million people with dementia around the world; this number will more than triple to 152 million by 2050 [1]. The worldwide cost of the disease was about US$ 1 trillion in 2018, and that will rise to US$ 2 trillion by 2030 [1]. The incidence of dementia in people aged 60 years old and over is 9.9 cases per 1000 person-years in China [2]. Based on estimation, the prevalence of dementia among individuals aged 65 years and older in China was around 5.1% [3], and are increasing nationwide. Due to the limited treatments available for dementia at present, primary prevention is the best strategy to reduce the risk or delay the onset of dementia in late life [4]. Mild cognitive impairment (MCI) is a key transitional state between normal aging and dementia [5]. Individuals with MCI have a higher risk of dementia, with a rate of 10–15% per year compared with the rate for healthy controls of 1–2% per year [6]. Therefore, identification of risk factors for MCI might be of significance to prevent dementia among older adults.

Body mass index (BMI) is considered one of the important factors affecting cognitive function. Previous studies found that unhealthy BMI was associated with cognitive function among older adults [7]. A cross-sectional study from Malaysia concluded positive association between BMI and cognitive function, measured by Mini-Mental State Examination (MMSE) in older adults aged 60 years and above [8]. Another cohort study of older blacks and whites (aged 53–100) conducted in the United States indicated that late-life BMI was related to change in cognitive function, with lower BMI being related to faster rates of decline in summary measures of global cognition [9]. Some studies have found that elevated BMI was associated with lower structural integrity in a brain region connecting frontal and temporal lobes among older people, which might affect their cognitive function [10, 11]. A retrospective cohort study found there was difference in associations between BMI, brain metabolism and connectivity in elderly females and males [12]. However, it is also not known whether there is a gender difference in the relationship between BMI and MCI.

A recent study indicated that higher BMI was associated with relatively poorer cognitive performance in older people aged 75 to 84, while the opposite relationship was found in older people over 84 years old [13]. Another study found that obesity was associated with a lower risk of cognitive decline among older people age 65 or older was found, but this protective association did not exist in adults aged 45 to 65 [14]. However, a study examined increased BMI might adversely affect cognitive function in older people aged 65 and over, but not in younger cohort [15]. The reason for the inconsistent results in different age group between BMI and cognition might be that age plays a moderating role.

Currently, the moderating role of gender and age in the relationship between BMI and MCI among Chinese older people is unclear. The percentage of people in China aged 60 years or above is 16.7% (231 million people) in 2016, which is expected to be more than 33.3% (491 million) in 2050, being about double of the percentage in 2016 [16]. About three-quarters of China’s elderly live in rural areas. Thus, the purpose of this study was to investigate the moderating effects of gender and age on the association between BMI and MCI among Chinese rural older adults.

## Methods

### Study setting and participants

This study used the data from the Health Service for Rural Elderly Families Survey in Shandong, which was conducted from May to June 2019 in Shandong province, China. There were more than 100 million people in Shandong in 2018, and people aged 60 and over accounted for 22.3% of the total population [17]. The population of Shandong also ranked the second among the total population of China [18], with largest number of elderly population. The multistage stratified cluster sampling method was used to select the subjects [19]. Firstly, according to the Gross Domestic Product (GDP) per capita in Shandong in 2018, we selected three rural counties (one above the medium level, Rushan from Weihai; one at the medium level, Qufu from Jining; and one below the medium level, Laoling from Dezhou) as study sites. Secondly, five townships were randomly selected from each rural county. Thirdly, we randomly selected four villages from each of the selected townships. Finally, older people aged or over 60 years old who were randomly selected from each sample village were recruited in the survey. Inclusion criteria were (1) permanent residents who lived in the village for over 6 months in the past year, and (2) aged 60 years old and above. Exclusion criterion were (1) respondents who had an inability to complete the interview or communicate with others, and (2) with a history of dementia by further asking the village doctors about the physical condition of the interviewees. All of the completed questionnaires were carefully checked by the supervisors after the interview each day. In total, 3600 individuals were recruited and 3243 completed the whole survey, with a response rate of 90.1%. Of the 3243 respondents, one respondent was excluded from analysis due to the lack of data in cognitive survey. Finally, a total of 3242 rural older adults were included in the analysis.

### Measures

#### Mild cognitive impairment

Mild cognitive impairment (MCI) was measured by the 30-item Chinese version of the Mini-Mental State Examination (MMSE). The maximum score is 30 points, with the higher scores indicating a better cognitive function. It showed good reliability and validity as an instrument for detecting MCI among Chinese [20–22]. The widely accepted cut-off point to determine MCI in China (MMSE Chinese Standard, MCS) based on education-specific are less than a score of 17 for respondents of illiteracy, less than a score of 20 for respondents of primary school, and less than a score of 24 for respondents of higher than primary school [23].

#### Body mass index

Body mass index (BMI) was calculated using the formula: BMI (kg/m^2^) = weight (kg) / height^2^ (m^2^). Height (in centimeter) was self-reported and weight (in kilogram) was measured by the interviewers. The self-reported height could underestimate overweight and obesity [24]. The same weight scale was used for all participants and the scale was calibrated before the measurement. BMI was categorized into underweight (BMI < 18.5 kg/m^2^, low BMI), normal weight (18.5 kg/m^2^ ≤ BMI < 24.0 kg/m^2^, normal BMI), overweight (24.0 kg/m^2^ ≤ BMI < 28.0 kg/m^2^, elevated BMI), and obesity (BMI ≥ 28.0 kg/m^2^, high BMI) according to the revised Asia-Pacific BMI criteria by the World Health Organization (WHO) [25].

#### Covariates

In this study, covariates included sociodemographic variables, health behavior and health status variables. Sociodemographic variables included age (< 75 or ≥ 75 years), gender (male or female), education (illiteracy, primary school, or junior high school or above), marital status (married or unmarried/widowed/divorced), and household income [quartile 1 (the poorest), quartile 2, quartile 3, or quartile 4 (richest)]. Health behavior variables included cigarette smoking (yes), alcohol consumption (yes), and physical activity (high level, moderate level, or low level). Health status variables included activities of daily livings (normal or impaired), mental health (good, general, worse, or bad), and chronic diseases (hypertension, diabetes, hyperlipidemia, coronary heart disease, and cerebrovascular disease). Participants reported whether they have been diagnosed with the above chronic conditions. Physical activity was measured by the Chinese version of the International Physical Activity Questionnaire Short Form (IPAQ-S) [26]. Activities of daily livings (ADL) were measured by the Instrumental Activity of Daily Living Scale (IADL). The score > 20 points indicates that there is a life activity disorder [27]. Level of mental health was measured by the Chinese version of the 10-item Kessler Psychological Distress Scale (K10). The total score ranges from 10 to 50 and can be divided into 4 groups: 10–15 indicating good mental health, 16–21 indicating general mental health, 22–29 indicating worse mental health, 30–50 indicating bad mental health [28].

#### Statistical analysis

We used Stata 16.0 to analyze the data. Firstly, descriptive statistics were used to describe baseline characteristics of participants with frequency (percentage). Pearson’s chi-square test for categorical variables was used to compare different groups without or with MCI. Then, since 3242 participants came from 60 villages and 2928 families, multilevel mixed-effects logistic regression was used to examine the moderating role of gender and age. Associations between BMI and MCI were also assessed by multilevel mixed-effects logistic regression. More details about multilevel mixed-effects logistic regression were described in Additional file 1. The odds ratio (OR) and 95% confidence interval (CI) were presented as measures of effect. In model 1, none covariates were included. In the literature [7, 29], we found that age, gender, education and other confounding factors may also affect the cognition of old people. Therefore, model 2 was based on model 1, with additional adjusting for the statistically significant (*p*-value< 0.05) covariates of the previous single factor analysis. We also used Firth method as a sensitivity analysis in Additional file 2. The statistically significant threshold was set at a two-sided and *p*-value< 0.05.

## Results

### Characteristics

Baseline characteristics of participants are shown in Table 1. Of 3242 participants, the prevalence of MCI defined by MMSE was 18.5%. The average age was 70.14 ± 6.17 years (range 60–100). About two-thirds of the participants were female. At baseline, 165 (5.1%) participants were low BMI. Among the participants with MCI, the mean BMI was 23.3 kg/m^2^ (SD: 3.9), while the mean BMI was 24.5 kg/m^2^ (SD: 3.9) in participants without MCI. There are some surprising findings here: the participants with MCI tended to be younger, female group, have not gone to school, with moderate physical exercise, without activity disorder, with good mental health. From Table 1, it doesn’t seem like there is a higher proportion of young people in the MCI group, but rather a higher proportion of old persons. The distribution of MMSE scores in the participants is presented in Additional file 3.

**Table 1:** Baseline characteristics according to cognitive function

Characteristics	N (%)	Mild cognitive impairment		***p-***valve
		No (%)	Yes (%)	
Total	3242 (100.00)	2641 (81.46)	601 (18.54)	
Age				**< 0.001**
< 75	2499 (77.08)	2127 (80.54)	372 (61.90)	
≥ 75	743 (22.92)	514 (19.46)	229 (38.10)	
Gender				**< 0.001**
Male	1181 (36.43)	1009 (38.21)	172 (28.62)	
Female	2061 (63.57)	1632 (61.79)	429 (71.38)	
Education				**< 0.001**
Illiteracy	1353 (41.73)	1005 (38.05)	384 (58.90)	
Primary school	1257 (38.77)	1107 (41.92)	150 (26.96)	
Junior high school or above	632 (19.49)	529 (20.03)	103 (17.14)	
Marital status				**< 0.001**
Married	2415 (74.49)	2065 (78.19)	350 (58.24)	
Unmarried/widowed/divorced	827 (25.51)	576 (21.81)	251 (41.76)	
Household income				**< 0.001**
Quartile 1 (the poorest)	816 (25.17)	589 (22.30)	227 (37.77)	
Quartile 2	803 (24.77)	660 (24.99)	143 (23.79)	
Quartile 3	809 (24.95)	692 (26.20)	117 (19.47)	
Quartile 4 (the richest)	814 (25.11)	700 (26.51)	114 (18.97)	
Cigarette smoking, yes	1003 (30.94)	846 (32.03)	157 (26.12)	**0.005**
Alcohol consumption, yes	922 (28.44)	782 (29.61)	140 (23.29)	**0.002**
Physical activity				**< 0.001**
High level	1322 (40.78)	1169 (44.26)	153 (25.46)	
Moderate level	1358 (41.89)	1074 (40.67)	284 (47.25)	
Low level	562 (17.33)	398 (15.07)	164 (27.29)	
ADL				**< 0.001**
Normal	2849 (87.88)	2383 (90.23)	466 (77.54)	
Impaired	393 (12.12)	258 (9.77)	135 (22.46)	
Mental health				**< 0.001**
Good	1845 (56.91)	1557(58.95)	288 (47.92)	
General	637 (19.65)	516(19.54)	121 (20.13)	
Worse	515 (15.89)	391(14.80)	124 (20.63)	
Bad	245 (7.55)	177(6.71)	68 (11.32)	
Hypertension, yes	1478 (45.59)	1206 (45.66)	272 (45.26)	0.857
Diabetes, yes	389 (12.00)	331 (12.53)	58 (9.65)	0.050
Hyperlipidemia, yes	146 (4.50)	123 (4.66)	23 (3.83)	0.376
Coronary heart disease, yes	572 (17.64)	468 (17.72)	104 (17.30)	0.809
Cerebrovascular disease, yes	202 (6.23)	160 (6.06)	42 (6.99)	0.395
BMI	24.31 (3.92)	24.54 (3.89)	23.29 (3.86)	**< 0.001**
Low BMI	165 (5.09)	105 (3.98)	60 (9.98)	
Normal BMI	1397 (43.09)	1107 (41.92)	290 (48.25)	
Elevated BMI	1160 (35.78)	972 (36.80)	188 (31.28)	
High BMI	520 (16.04)	457 (17.30)	63 (10.49)	
MMSE scores, Mean (SD)	22.91 (5.11)	24.63 (3.54)	15.35 (3.90)	**< 0.001**
2–17	406 (12.52)	–	406 (67.55)	
18–20	372 (11.47)	251 (9.50)	121 (20.13)	
21–24	759 (23.41)	685 (25.94)	74 (12.31)	
25–30	1705 (52.59)	1705 (64.56)	–	

*ADL* activities of daily livings, *BMI* body mass index, *MMSE* Mini-Mental State Examination, *SD* standard deviation

### Moderating effects of gender and age on the association between BMI and MCI

**Fig. 1:**
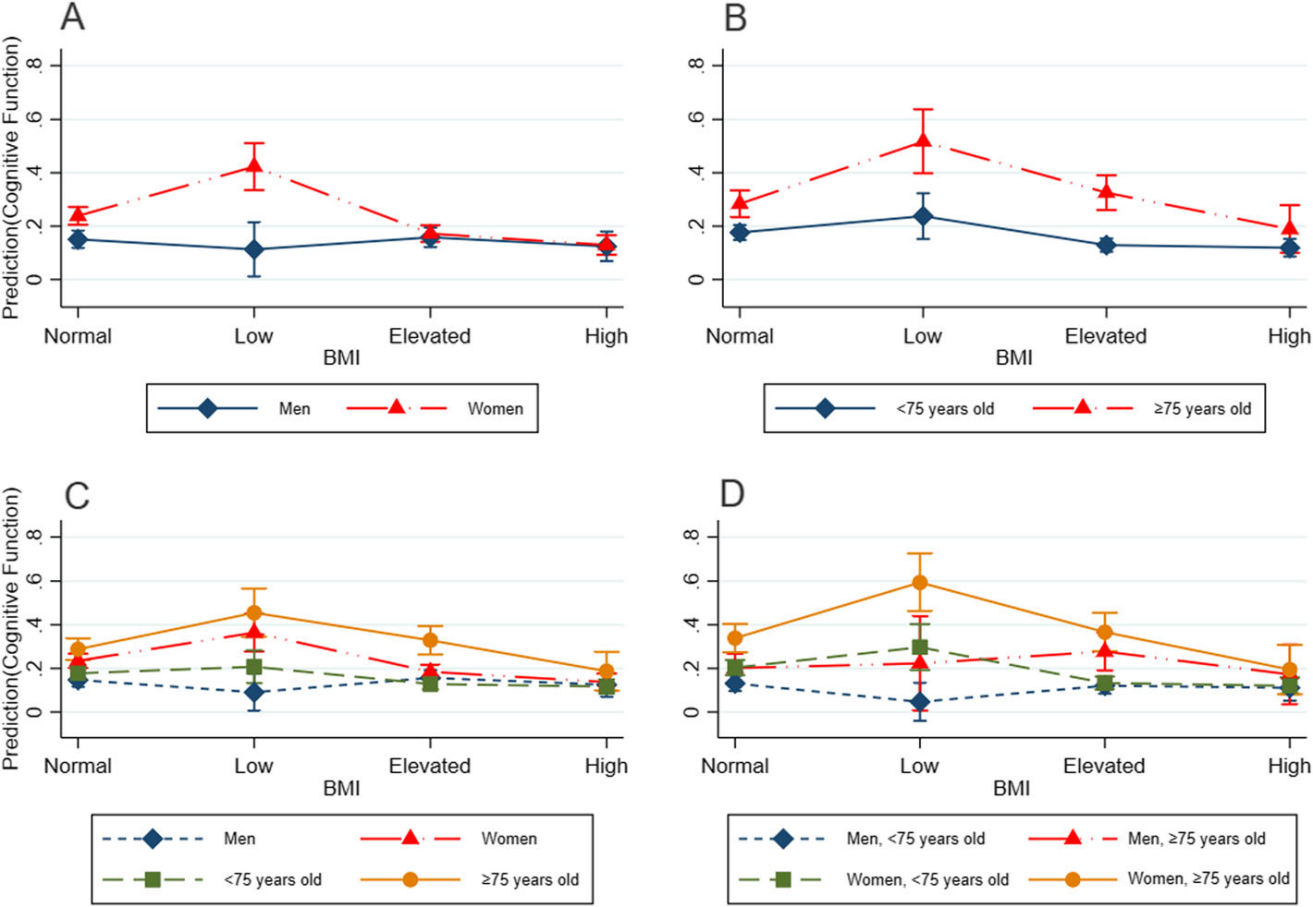
Interaction between BMI, gender and age in the prediction of cognitive function with unadjusted 95% confidence interval based on the model 1. BMI, body mass index. **a** represents BMI × gender. **b** represents BMI × age. **c** represents BMI × gender and BMI × age. **d** represents BMI × gender×age

**Fig. 2:**
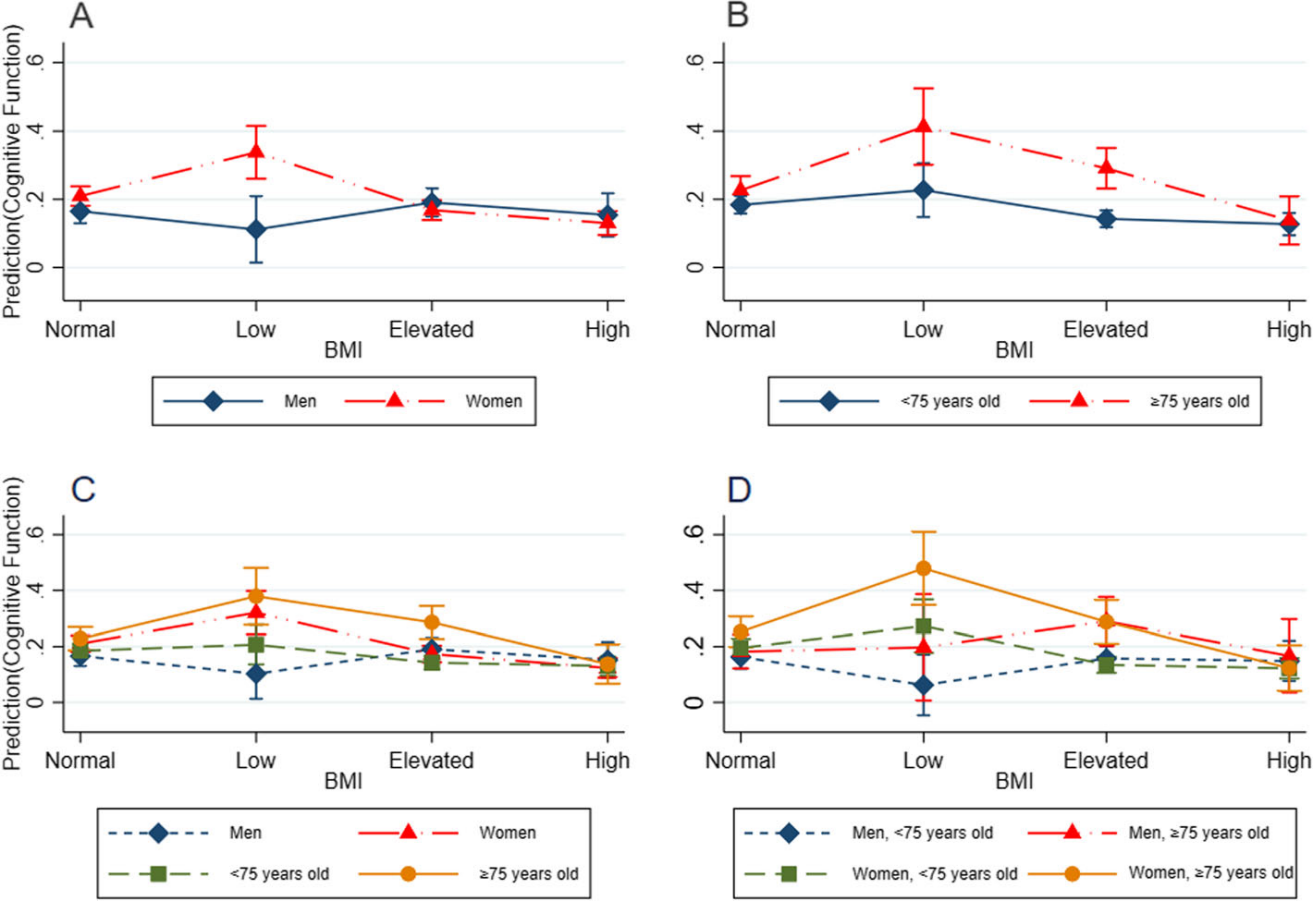
Interaction between BMI, gender and age in the prediction of cognitive function with adjusted 95% confidence interval based on the model 2. BMI, body mass index. Model 2 was adjusted for age, gender, marital status, household income, cigarette, alcohol consumption, physical activity, activities of daily livings, mental health. **a** represents BMI × gender. **b** represents BMI × age. **c** represents BMI × gender and BMI × age. **d** represents BMI × gender×age

Figure 1 shows the interaction between BMI, gender and age in the prediction of cognitive function with unadjusted 95% CI based on the model 1. There were significant interactions in BMI × gender, BMI × age with MCI in low BMI and women group, as well as elevated BMI and older elderly group. When exploring joint interactions of BMI × gender and BMI × age with MCI was statistically significant in elevated BMI and older elderly group, as well as low BMI, women and older elderly group. Figure 2 shows the interaction between BMI, gender and age in the prediction of cognitive function with adjusted 95% CI based on the model 2. After adjusted for covariates, the interactions of each group were still statistically significant.

### Association between BMI and MCI in older adults and subgroups

Table 2 presents the relationship between BMI and MCI among older people and subgroups. Among whole participants, low BMI was associated with a higher risk of MCI (aOR = 2.08, 95% CI: 1.26–3.44, *p* < 0.01), and high BMI was correlated with a lower risk of MCI (aOR = 0.55, 95% CI: 0.37–0.82, *p* < 0.01) when compared to normal BMI. Low BMI was also associated with a higher risk of MCI (aOR = 2.06, 95% CI: 1.35–3.12, *p* < 0.01), and high BMI was correlated with a lower risk of MCI (aOR = 0.52, 95% CI: 0.36–0.75, *p* < 0.01) only in women. In the younger elderly (< 75 years old), participants with elevated BMI (aOR = 0.66, 95% CI: 0.47–0.93, *p* < 0.05) and high BMI (aOR = 0.53, 95% CI: 0.33–0.84, *p* < 0.01) were less likely to have MCI. On the contrary, participants with low BMI had higher odds of MCI (aOR = 3.20, 95% CI: 1.34–7.45, *p* < 0.01) in the older elderly (≥75 years old).

**Table 2:** Association between BMI and MCI in older adults and subgroups. Values are odds ratio (95% confidence interval)

BMI	All (***n*** = 3242)		Men (***n*** = 1181)		Women (***n*** = 2061)		Aged 60–74 (***n*** = 2499)		Aged 75–100 (***n*** = 743)	
	Model 1	Model 2	Model 1	Model 2	Model 1	Model 2	Model 1	Model 2	Model 1	Model 2
Low BMI	2.72*** (1.66–4.43)	2.08** (1.26–3.44)	0.68 (0.23–1.98)	0.57 (0.18–1.77)	2.41*** (1.63–3.57)	2.06** (1.35–3.12)	1.56 (0.84–2.91)	1.48 (0.74–2.98)	3.65** (1.60–8.31)	3.20** (1.34–7.45)
Normal BMI	1.00 (reference)	1.00 (reference)	1.00 (reference)	1.00 (reference)	1.00 (reference)	1.00 (reference)	1.00 (reference)	1.00 (reference)	1.00 (reference)	1.00 (reference)
Elevated BMI	0.73* (0.56–0.95)	0.86 (0.65–1.12)	1.03 (0.72–1.46)	1.24 (0.84–1.81)	0.64** (0.50–0.83)	0.73* (0.56–0.96)	0.63** (0.46–0.86)	0.66* (0.47–0.93)	1.25 (0.78–1.99)	1.65 (0.97–2.82)
High BMI	0.50*** (0.34–0.73)	0.55** (0.37–0.82)	0.74 (0.42–1.30)	0.91 (0.50–1.65)	0.47*** (0.33–0.67)	0.52** (0.36–0.75)	0.55** (0.37–0.84)	0.53** (0.33–0.84)	0.51 (0.23–1.14)	0.44 (0.18–1.07)

Model 1 was unadjusted

Model 2 was adjusted for age, gender, education, marital status, household income, cigarette, alcohol consumption, physical activity, activities of daily livings, mental health

*BMI* body mass index, *MCI* mild cognitive impairment

*p < 0.05; **p < 0.01; ***p < 0.001

### Association between BMI and MCI in four subgroups by gender and age

Results of association between BMI and MCI in four subgroups divided by gender and age are shown in Table 3. Comparing to normal BMI, elevated BMI was correlated with a higher risk of MCI (aOR = 2.32, 95% CI: 1.17–4.61, *p* < 0.05) among older men. Among younger women, elevated BMI (aOR = 0.60, 95% CI: 0.44–0.83, *p* < 0.01) and high BMI (aOR = 0.52, 95% CI: 0.35–0.79, *p* < 0.01) were associated with a lower risk of MCI. Among the older women, low BMI was associated with a higher risk of MCI (aOR = 3.38, 95% CI: 1.69–6.73, *p* < 0.01). High BMI remained related to a lower risk of MCI (aOR = 0.39, 95% CI: 0.16–0.93, *p* < 0.05) among older women. An analysis of a non-linear association between BMI and MMSE score with stratification on gender and age is shown in Additional file 4.

**Table 3:** Association between BMI and MCI in four subgroups by gender and age. Values are odds ratio (95% confidence interval)

BMI	Men (***n*** = 1181)				Women (***n*** = 2061)			
	Aged 60–74 (***n*** = 883)		Aged 75–100 (***n*** = 298)		Aged 60–74 (***n*** = 1616)		Aged 75–100 (***n*** = 445)	
	Model 1	Model 2	Model 1	Model 2	Model 1	Model 2	Model 1	Model 2
Low BMI	0.29 (0.04–2.19)	0.28 (0.03–2.34)	1.10 (0.29–4.22)	1.25 (0.28–5.56)	1.66 (0.96–2.87)	1.60 (0.91–2.81)	3.03** (1.61–5.73)	3.38** (1.69–6.73)
Normal BMI	1.00 (reference)	1.00 (reference)	1.00 (reference)	1.00 (reference)	1.00 (reference)	1.00 (reference)	1.00 (reference)	1.00 (reference)
Elevated BMI	0.87 (0.57–1.35)	0.97 (0.60–1.56)	1.51 (0.83–2.75)	2.32* (1.17–4.61)	0.57*** (0.42–0.78)	0.60** (0.44–0.83)	1.10 (0.67–1.79)	1.30 (0.76–2.23)
High BMI	0.73 (0.37–1.42)	0.81 (0.40–1.67)	0.81 (0.28–2.29)	1.22 (0.36–4.08)	0.50** (0.34–0.75)	0.52** (0.35–0.79)	0.46 (0.21–1.04)	0.39* (0.16–0.93)

Model 1 was unadjusted

Model 2 was adjusted for age, gender, education, marital status, household income, cigarette, alcohol consumption, physical activity, activities of daily livings, mental health

*BMI* body mass index, *MCI* mild cognitive impairment

*p < 0.05; **p < 0.01; ***p < 0.001

## Discussion

In this study, we found the prevalence of MCI among rural older people was 18.5%. The prevalence in present study was higher than older adults (60+) in Shanghai suburb (by MMSE, 7.0%) [30], and older people (65+) from 8 longevity areas of China (by MMSE, 10.0%) [31]. A survey of community-based cohort in Zhejiang Province showed that the prevalence of MCI (by MMSE) among older adults (60+) was 14.7% [7]. The prevalence of MCI among older people in previous studies was lower than that in present study. This may be because that we only focused on the older adults in rural areas, and previous studies have indicated that the prevalence of MCI in rural areas was significantly higher than that in urban areas [32].

The moderating effect of gender between BMI and MCI was found. Similar to previous studies, older women with low BMI were more likely to have MCI and those with elevated or high BMI were less likely to have MCI. However, the relationships were not significant in males. Malnutrition could be a risk factor for cognitive decline among older people [33]. BMI is an important objective indicator assessing the nutritional status among older people. Low BMI might be related to malnutrition. One possible explanation for the observed gender difference might be that differences in estrogen levels. Estrogen is a group of steroid compounds that function as primary gender hormones in women [34]. Estrogen interacts with different types of receptors to exerted its action, including estrogen receptors ER-α and ER-β that are highly expressed in the brain [35]. In men, testosterone is converted to estradiol by aromatase enzyme, which is located around and throughout the male brain [36]. Since the secretion of testosterone has never completely stopped, the serum estrogen levels are higher among older men than in postmenopausal older women. Studies have shown that sex steroids such as estrogen and testosterone could protect the brain’s function during aging [37]. Thus, different levels of steroids in men and women may affect their cognitive function to varying degrees.

The moderating effect of age between BMI and MCI was found. Different from previous studies, low BMI was positively correlated with MCI in older elderly, and elevated or high BMI was negative correlated with MCI in younger elderly. Respecting the differences between younger and older elderly, a possible explanation was that older elderly became more frail as they got older and the frailty accelerated increases. Frailty in older people were predisposed to a homeostatic failure of complex systems [38], which could lead to more functional deficits. In Chinese rural areas, the degree of frailty of older people remained at a high level, and more than half of elderly households were moderate and advanced frailty [39]. Studies have found that the prevalence of cognitive impairment in the frail elderly was higher than that of non-frail elderly [40, 41]. Another possible reason was that the overall BMI decreased with age among older people. Lower BMI might affect brain atrophy through changes in metabolic and inflammatory [42]. Another important reason might be related to alterations in body composition. Aging was characterized by the decrease of lean body mass, and higher lean body mass might reduce the risk of cognitive impairment among older people [43], which could contribute to a lower risk of development of MCI in younger elderly with elevated or high BMI.

Inconsistent with previous studies, we found elevated BMI was positively correlated with MCI in older male elderly. Previous studies from South Korea [14, 44], Australia [45], and the United States [13] have found that higher BMI was not associated with decreased cognitive function or with better cognitive performance among older men. Amyloid-beta protein produced by amyloid precursor protein (app) cleavage could form amyloid aggregates and has long been considered the culprit for Alzheimer’s [46]. A recent study found that dysregulation of app impairs adipose tissue mitochondrial function and promotes obesity [47]. Therefore, the association between elevated BMI and MCI in older male elderly might be due to the common pathological mechanism: abnormal amyloid precursor protein. However, the gender and age differences need to be further studied.

Moreover, we further found this association between low BMI and MCI became stronger among older women. It had been shown previously that older adults who had low BMI or lost weight were predisposed to cognitive decline. Previous studies from China [30, 31], South Korea [44], and the United States [48] differing from the results of this study have found only that underweight was associated with MCI in women or older adults in general. We should pay more attention to the relationship between low BMI and cognitive function among older women.

The findings demonstrated the importance of managing the weight of rural older adults by gender and age. Government departments should strengthen the health management of malnutrition or low BMI for the rural older people, especially in the older female elderly. We recommend strengthening the information management of BMI and cognitive function among rural older adults. We suggest combine the basic public health services for the health management among older people to improve their understanding of the healthy weight. By grouping the weight of older people, high-risk groups with impaired cognitive function can be detected early to prevent the risk of dementia and maintain the well-being of older adults.

The current study had several limitations. First, waist circumference was not included to assess elevated or high BMI among older people; we will include this indicator in the following study. We would use a portable stadiometer to measure height objectively for the future research. Second, we could not determine the causal relationship between BMI and MCI as the cross-sectional data we used, longitudinal studies are needed in the future to explore the causal relationships.

## Conclusions

This study demonstrated that the relationship between BMI and MCI was moderated by gender and age. Older adults, especially older women with a low BMI, had a higher risk of MCI, compared to the normal BMI. Older men with elevated BMI more likely to have MCI. Government departments should strengthen the health management of rural elderly with malnutrition or low BMI, especially older women.

## Supplementary Information

**Additional file 1.** An introduction of multilevel mixed-effects logistic regression

**Additional file 2.** A sensitivity analysis

**Additional file 3: Figure S1.** Distribution of MMSE scores among older adults

**Additional file 4.** An analysis of a non-linear association

## Abbreviations

BMI: Body mass index
MCI: Mild cognitive impairment
MMSE: Mini-Mental State Examination
