# Supplementary material for: Body mass index and mild cognitive impairment among rural older adults in China: the moderating roles of gender and age
Source: BMC Psychiatry. 2021 Jan 23;21:54. doi: 10.1186/s12888-021-03059-8 (PMC7825154; doi:10.1186/s12888-021-03059-8)
Supplement: Supplementary file 2 — Additional file 2. A sensitivity analysis [file 12888_2021_3059_MOESM2_ESM.doc]

**Additional file 2**

**A sensitivity analysis**

Using logistic regression might pose a small sample (low event) bias in this study. Because there were only 5% of the participants with low body mass index (BMI). We try Firth method to overcome this bias as a sensitivity analysis. Also called penalized likelihood , the Firth method is a general approach to reducing small-sample bias in maximum likelihood estimation [1]. The results, as shown in Table S1 and Table S2, are not substantially different from the use of multilevel mixed-effect logistic regression.

**References**

1. Williams R. Analyzing Rare Events with Logistic Regression. University of Notre Dame. 2019. https://www3.nd.edu/~rwilliam/stats3/RareEvents.pdf

**Table S1 Association between BMI and MCI in older adults and subgroups. Values are odds ratio (95% confidence interval)**

| **BMI** | **All (n=3242)** | |  | **Men (n=1181)** | |  | **Women (n=2061)** | |  | | **Aged 60-74 (n=2499)** | |  | **Aged 75-100 (n=743)** | |
| --- | --- | --- | --- | --- | --- | --- | --- | --- | --- | --- | --- | --- | --- | --- | --- |
|  | **Model 1** | **Model 2** |  | **Model 1** | **Model 2** |  | **Model 1** | **Model 2** |  | **Model 1** | | **Model 2** |  | **Model 1** | **Model 2** |
| Low BMI | **2.19*****  **(1.55-3.08)** | **1.77****  **(1.23-3.57)** |  | 0.73  (0.27-2.01) | 0.63  (0.21-1.83) |  | **2.44*****  **(1.66-3.57)** | **2.06****  **(1.36-3.10)** |  | 1.47  (0.90-2.40) | | 1.35  (0.81-2.25) |  | **2.86*****  **(1.70-4.81)** | **2.59****  **(1.47-4.55)** |
| Normal BMI | 1.00  (reference) | 1.00  (reference) |  | 1.00  (reference) | 1.00  (reference) |  | 1.00  (reference) | 1.00  (reference) |  | 1.00  (reference) | | 1.00  (reference) |  | 1.00  (reference) | 1.00  (reference) |
| Elevated BMI | **0.74****  **(0.60-0.90)** | 0.87  (0.70-1.07) |  | 1.02  (0.72-1.45) | 1.26  (0.87-1.82) |  | **0.62*****  **(0.48-0.80)** | **0.73***  **(0.56-0.95)** |  | **0.66****  **(0.51-0.85)** | | **0.71****  **(0.55-0.92)** |  | 1.15  (0.80-1.64) | 1.46  (0.99-2.17) |
| High BMI | **0.53*********  **(0.39-0.71)** | **0.59****  **(0.44-0.81)** |  | 0.74  (0.43-1.28) | 0.93  (0.52-1.66) |  | **0.44*****  **(0.31-0.62)** | **0.52*****  **(0.36-0.74)** |  | **0.57****  **(0.41-0.80)** | | **0.58****  **(0.41-0.83)** |  | 0.58  (0.31-1.06) | 0.53  (0.27-1.02) |

**p* < 0.05; ***p* < 0.01; ****p* < 0.001.

Note: BMI, body mass index, MCI, mild cognitive impairment.

Model 1 was unadjusted.

Model 2 was adjusted for age, gender, education, marital status, household income, cigarette, alcohol consumption, physical [activity](../../../../D:/Program%20Files%20(x86)/Dict/8.5.3.0/resultui/html/index.html#/javascript:;), activities of daily livings, mental health.

**Table S2 Association between BMI and MCI in four subgroups by gender and age. Values are odds ratio (95% confidence interval)**

| **BMI** | **Men (n=1181)** | | | | |  | **Women (n=2061)** | | | | |
| --- | --- | --- | --- | --- | --- | --- | --- | --- | --- | --- | --- |
|  | **Aged 60-74 (n=883)** | |  | **Aged 75-100 (n=298)** | |  | **Aged 60-74 (n=1616)** | |  | **Aged 75-100 (n=445)** | |
|  | **Model 1** | **Model 2** |  | **Model 1** | **Model 2** |  | **Model 1** | **Model 2** |  | **Model 1** | **Model 2** |
| Low BMI | 0.41  (0.08-2.21) | 0.39  (0.06-2.39) |  | 1.21  (0.34-4.28) | 1.35  (0.35-5.21) |  | 1.69  (0.99-2.88) | 1.61  (0.87-2.74) |  | **3.01*****  **(1.65-5.52)** | **3.19****  **(1.65-6.16)** |
| Normal BMI | 1.00  (reference) | 1.00  (reference) |  | 1.00  (reference) | 1.00  (reference) |  | 1.00  (reference) | 1.00  (reference) |  | 1.00  (reference) | 1.00  (reference) |
| Elevated BMI | 0.88  (0.57-1.35) | 1.00  (0.63-1.58) |  | 1.50  (0.84-2.70) | **2.16***  **(1.14-4.11)** |  | **0.57*****  **(0.42-0.77)** | **0.61****  **(0.44-0.83)** |  | 1.03  (0.65-1.63) | 1.26  (0.76-2.11) |
| High BMI | 0.76  (0.39-1.45) | 0.87  (0.44-1.73) |  | 0.86  (0.32-2.34) | 1.27  (0.41-3.91) |  | **0.49*****  **(0.33-0.73)** | **0.53****  **(0.35-0.79)** |  | 0.48  (0.22-1.02) | **0.43***  **(0.19-0.98)** |

**p* < 0.05; ***p* < 0.01; ****p* < 0.001.

Note: BMI, body mass index, MCI, mild cognitive impairment.

Model 1 was unadjusted.

Model 2 was adjusted for age, gender, education, marital status, household income, cigarette, alcohol consumption, physical [activity](../../../../D:/Program%20Files%20(x86)/Dict/8.5.3.0/resultui/html/index.html#/javascript:;), activities of daily livings, mental health.
