# Supplementary material for: Body mass index and mild cognitive impairment among rural older adults in China: the moderating roles of gender and age
Source: BMC Psychiatry. 2021 Jan 23;21:54. doi: 10.1186/s12888-021-03059-8 (PMC7825154; doi:10.1186/s12888-021-03059-8)
Supplement: Supplementary file 3 — Additional file 3: Figure S1. Distribution of MMSE scores among older adults [file 12888_2021_3059_MOESM3_ESM.doc]

**Additional file 3**


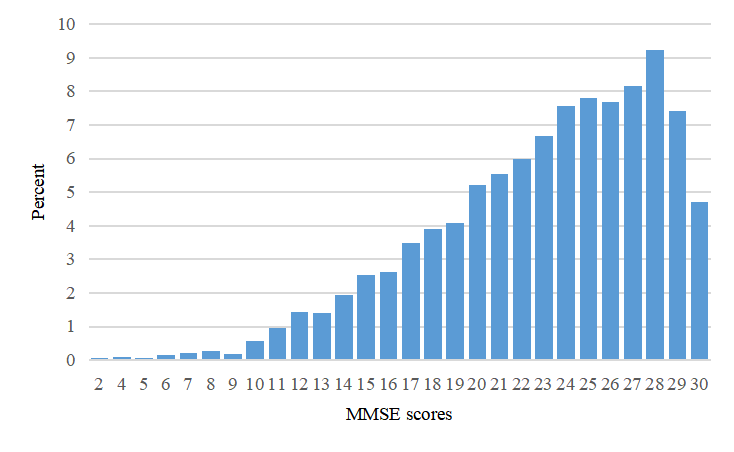


**Figure S1** **Distribution of MMSE scores among older adults (N=3,242)**. MMSE, Mini-Mental State Examination.
