# Supplementary material for: Body mass index and mild cognitive impairment among rural older adults in China: the moderating roles of gender and age
Source: BMC Psychiatry. 2021 Jan 23;21:54. doi: 10.1186/s12888-021-03059-8 (PMC7825154; doi:10.1186/s12888-021-03059-8)
Supplement: Supplementary file 4 — Additional file 4. An analysis of a non-linear association [file 12888_2021_3059_MOESM4_ESM.doc]

**Additional file 4**

**An analysis of a non-linear association**

A non-linear association was found in this study. An analysis of a non-linear association between body mass index (BMI) and Mini-Mental State Examination (MMSE) score with stratification on gender and age is shown here. Initially, we combined box plots and scatter plots to remove 4 samples of outliers, and finally included 3238 samples. Firstly, we made a scatter plot and added a fitting line for BMI and MMSE score, and the result is shown in figure S2.We found that there may be a quadratic nonlinear relationship between BMI and cognitive scores. Secondly, multiple nonlinear regression analysis was used to explore the relationship between BMI and MMSE score. Table S3 and table S4 present the results of multivariate analysis.


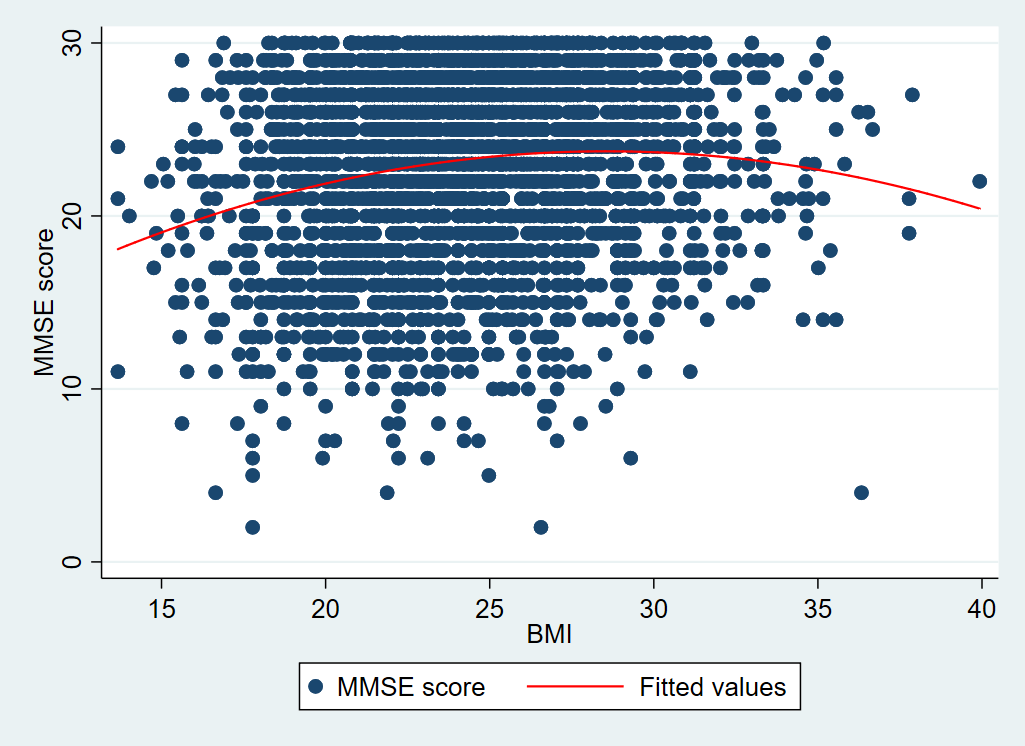


**Figure** **S2 A scatter plot of BMI and MMSE score (N=3238).** BMI, body mass index, MMSE, Mini-Mental State Examination.

**Table S3 Association between BMI and MMSE score in older adults and subgroups**

|  | **All (n=3238)** | | |  | **Men (n=1178)** | | |  | **Women (n=2060)** | | |  | **Aged 60-74 (n=2497)** | | |  | **Aged 75-100 (n=741)** | | |
| --- | --- | --- | --- | --- | --- | --- | --- | --- | --- | --- | --- | --- | --- | --- | --- | --- | --- | --- | --- |
|  | **B** | **SE** | ***p*-value** |  | **B** | **SE** | ***p*-value** |  | **B** | **SE** | ***p*-value** |  | **B** | **SE** | ***p*-value** |  | **B** | **SE** | ***p*-value** |
| BMI | 0.098 | 0.019 | **<0.001** |  | 0.144 | 0.315 | 0.649 |  | 0.745 | 0.220 | **0.001** |  | 0.566 | 0.203 | **0.005** |  | 0.400 | 0.412 | 0.331 |
| BMI^2 | -1.195 | 0.184 | **<0.001** |  | -0.002 | 0.006 | 0.723 |  | -0.013 | 0.004 | **0.004** |  | -0.010 | 0.004 | **0.015** |  | -0.005 | 0.009 | 0.545 |
| R^2^ | 0.361 | | |  | 0.303 | | |  | 0.345 | | |  | 0.326 | | |  | 0.373 | | |

Note: BMI, body mass index, MMSE, Mini-Mental State Examination, B, beta, SE, standard error.

Models were adjusted for age, gender, education, marital status, household income, cigarette, alcohol consumption, physical [activity](../../../../D:/Program%20Files%20(x86)/Dict/8.5.3.0/resultui/html/index.html#/javascript:;), activities of daily livings, mental health.

**Table S4 Association between BMI and MMSE score in in four subgroups by gender and age**

|  | **Men (n=1178)** | | | | | | |  | **Women (n=2060)** | | | | | | |
| --- | --- | --- | --- | --- | --- | --- | --- | --- | --- | --- | --- | --- | --- | --- | --- |
|  | **Aged 60-74 (n=882)** | | |  | **Aged 75-100 (n=296)** | | |  | **Aged 60-74 (n=1615)** | | |  | **Aged 75-100 (n=445)** | | |
|  | **B** | **SE** | ***p*-value** |  | **B** | **SE** | ***p*-value** |  | **B** | **SE** | ***p*-value** |  | **B** | **SE** | ***p*-value** |
| BMI | 0.130 | 0.353 | 0.713 |  | 0.320 | 0.668 | 0.633 |  | 0.734 | 0.250 | **0.003** |  | 0.312 | 0.529 | 0.556 |
| BMI^2 | -0.002 | 0.007 | 0.815 |  | -0.007 | 0.014 | 0.75833 |  | -0.013 | 0.005 | **0.009** |  | -0.002 | 0.011 | 0.865 |
| R^2^ | 0.305 | | |  | 0.290 | | |  | 0.289 | | |  | 0.385 | | |

Note: BMI, body mass index, MMSE, Mini-Mental State Examination, B, beta, SE, standard error.

Models were adjusted for age, gender, education, marital status, household income, cigarette, alcohol consumption, physical [activity](../../../../D:/Program%20Files%20(x86)/Dict/8.5.3.0/resultui/html/index.html#/javascript:;), activities of daily livings, mental health.
